# Chlorido{5-chloro-2-[2-(methyl­sulfanyl)phenyl­diazen­yl]phenyl}­platinum(II)

**DOI:** 10.1107/S1600536808000767

**Published:** 2008-01-16

**Authors:** Vivek Bagchi, Debkumar Bandyopadhyay

**Affiliations:** a Department of Chemistry, Indian Institute of Technology, New Delhi 110 016, India

## Abstract

The title compound, [Pt(C_13_H_10_ClN_2_S)Cl], contains a Pt atom tetra­coordinated by a benzene C, a diazene N, a Cl and an S atom in an approximately square-planar geometry. The mol­ecules dimerize through a nonbonded S⋯S inter­action [S⋯S = 3.523 (18) Å]. There are no hydrogen bonds and the crystal packing is stabilized by four inter­molecular π–π inter­actions; the centroid–centroid distances are 3.804 (3), 3.638 (3), 3.804 (3) and 3.638 (3) Å, and the corresponding perpendicular distances are 3.369, 3.448, 3.406 and 3.466 Å.

## Related literature

For related literature, see: Bagchi *et al.* (2007 ▶); Chattopadhyay *et al.* (1991 ▶); Dupont *et al.* (2005 ▶); Buraway & Vellins (1954 ▶).
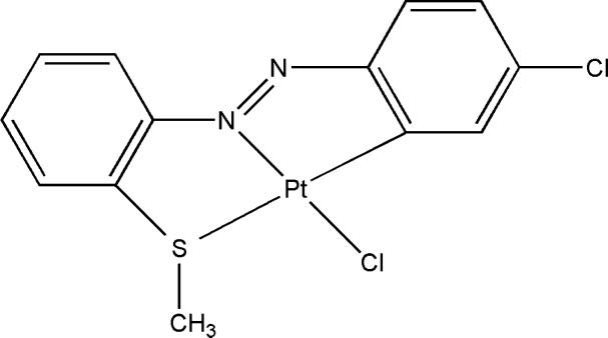


## Experimental

### 

#### Crystal data

[Pt(C_13_H_10_ClN_2_S)Cl]
*M* *_r_* = 492.28
Triclinic, 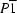

*a* = 7.424 (2) Å
*b* = 8.777 (3) Å
*c* = 11.069 (3) Å
α = 105.428 (4)°
β = 91.798 (4)°
γ = 96.641 (4)°
*V* = 689.1 (4) Å^3^
*Z* = 2
Mo *K*α radiation
μ = 10.70 mm^−1^
*T* = 273 (2) K
0.42 × 0.28 × 0.19 mm

#### Data collection

Bruker SMART CCD area-detector diffractometer
Absorption correction: multi-scan (*SADABS*; Sheldrick, 1996 ▶) *T*_min_ = 0.035, *T*_max_ = 0.134
6783 measured reflections
2544 independent reflections
2472 reflections with *I* > 2σ(*I*)
*R*_int_ = 0.025

#### Refinement

*R*[*F* ^2^ > 2σ(*F* ^2^)] = 0.018
*wR*(*F* ^2^) = 0.043
*S* = 1.05
2544 reflections
173 parameters
H-atom parameters constrained
Δρ_max_ = 0.89 e Å^−3^
Δρ_min_ = −0.89 e Å^−3^

### 

Data collection: *SMART* (Bruker, 1998 ▶); cell refinement: *SAINT* (Bruker, 2000 ▶); data reduction: *SAINT*; program(s) used to solve structure: *SHELXS97* (Sheldrick, 2008 ▶); program(s) used to refine structure: *SHELXL97* (Sheldrick, 2008 ▶); molecular graphics: *SHELXTL* (Sheldrick, 2008 ▶); software used to prepare material for publication: *SHELXTL*.

## Supplementary Material

Crystal structure: contains datablocks I, global. DOI: 10.1107/S1600536808000767/at2531sup1.cif

Structure factors: contains datablocks I. DOI: 10.1107/S1600536808000767/at2531Isup2.hkl

Additional supplementary materials:  crystallographic information; 3D view; checkCIF report

## Figures and Tables

**Table:** 

Pt1—N1	1.959 (3)
Pt1—C12	1.986 (3)
Pt1—Cl1	2.2911 (11)
Pt1—S1	2.3529 (10)

**Table:** 

N1—Pt1—C12	79.26 (14)
C12—Pt1—Cl1	96.84 (11)
N1—Pt1—S1	86.25 (9)
Cl1—Pt1—S1	97.57 (4)

**Fig. 1.**
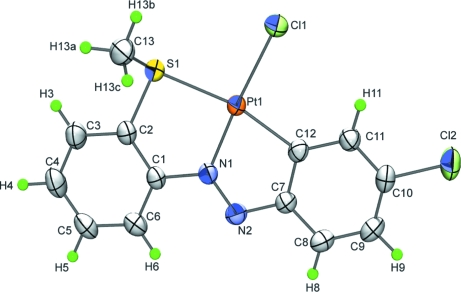
The asymmetric unit of (I), with displacement ellipsoids drawn at the 50% probability level.

**Fig. 2.**
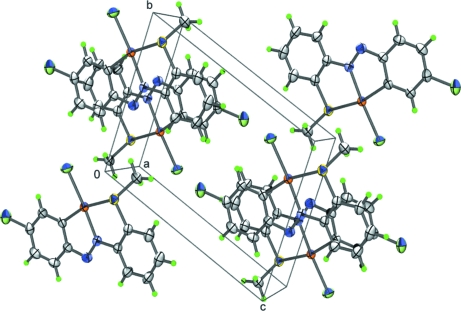
The molecular arrangement of (I) in the unitcell.

**Fig. 3.**
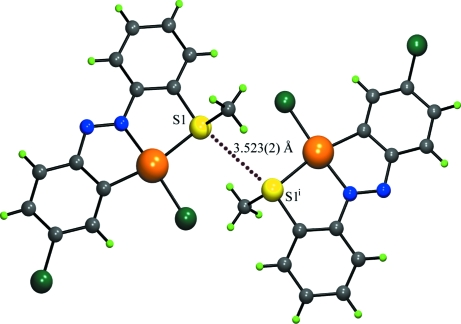
The intermolecular S···S interaction for (I), indicated by dotted line [symmetry code: (i) -x, 2 - y, -z].

**Fig. 4.**
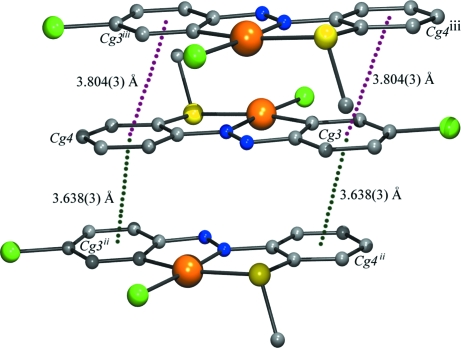
The intermolecular π—π interactions for (I), indicated by the dotted lines. [Symmetry codes: (ii) -x, 1 - y, -z, (iii) 1 - x, 1 - y, -z]. Cg3 and Cg4 are centroids of C1—C6 and C7—C12 rings, respectively.

## supplementary crystallographic information

### Comment

Cyclometallated compounds have numerous applications (Dupont *et al.*, 2005) in organic synthesis, catalysis and metallomesogen chemistry. Although a number of cycloplatinated complexes have been reported in the literature, report of platinum complexes with sulfur as auxiliary donor and the existence of C–Pt bond are sparse. Herein we report the crystal structure of (I) having such features.

The molecular structure of the title compound, (I), is shown in Fig. 1, with the atom numbering scheme. The platinum atom along with donor set of four atoms lie almost in one plane. Selected bond lengths, bond angles are listed in Table 1. The packing arrangement of (I) is shown in Fig. 2. The N?N bond length is similar of other cycloplatinated azoarenes (Chattopadhyay *et al.*, 1991).

The metal carbon bond length, 1.986 (3) Å, is slightly lower than the reported values of other *ortho*-metallated azoarenes (Chattopadhyay *et al.*, 1991). The molecules are found to dimerize through non-bonded S···S interaction; having S···S^i^ [symmetry code: (i) -*x*, 2 - *y*, -*z*] distance of 3.523 (18) Å (Bagchi *et al.* 2007) (Fig.3).The crystal packing is stabilized by four inter-molecular π-π interactions; the *Cg3*—*Cg4*^ii^, *Cg3*—*Cg4*^iii^, *Cg4*—*Cg3*^ii^, *Cg4*—*Cg3*^iii^, [symmetry codes: (ii) -*x*, 1 - *y*, -*z*, (iii) 1 - *x*, 1 - *y*, -*z. Cg3 *and *Cg4* are the centroids of C1—C6 and C7—C12 rings, respectively] distances are 3.638 (3), 3.804 (3), 3.638 (3) and 3.804 (3) Å, respectively; the corresponding perpendicular distances are 3.448, 3.369, 3.466 and 3.406 Å, respectively (Fig. 4).

### Experimental

2-(Methylsulfanyl)diazenyl-4-chlorobenzene was prepared by coupling 2-(methylsulfanyl)aniline with 4-nitroso-chlorobenzene. The ligand thus obtained was reacted with K_2_PtCl_4_ following a reported method (Buraway & Vellins, 1954). The product was purified by column chromatographic technique using silica gel column and methanol and dichloromethane (1:9 *v/v*) mixture as eluent. The solvent was evaporated in vacuum to obtain the pure product (78.3%). Suitable crystals of (I) were grown from a dichloromethane–hexane solution by slow evaporation.

### Refinement

H atoms were included at calculated positions as riding atoms with C—H set to 0.93 Å for (aromatic) and 0.96 Å for (CH_3_) H atoms, with *U*_iso_(H) = 1.2*U*_eq_(C) [1.5*U*_eq_ for methyl group].

### Crystal data

**Table:**

[Pt(C_13_H_10_ClN_2_S)Cl]	*Z* = 2
*M**_r_* = 492.28	*F*_000_ = 460.0
Triclinic, *P*1	*D*_x_ = 2.372 Mg m^−^^3^
Hall symbol: -P 1	Mo *K*α radiation λ = 0.71073 Å
*a* = 7.424 (2) Å	Cell parameters from 2472 reflections
*b* = 8.777 (3) Å	θ = 1.0–25.5º
*c* = 11.069 (3) Å	µ = 10.70 mm^−^^1^
α = 105.428 (4)º	*T* = 273 (2) K
β = 91.798 (4)º	Block, pink
γ = 96.641 (4)º	0.42 × 0.28 × 0.19 mm
*V* = 689.1 (4) Å^3^	

### Data collection

**Table:**

Bruker SMART CCD area-detector diffractometer	2544 independent reflections
Radiation source: fine-focus sealed tube	2472 reflections with *I* > 2σ(*I*)
Monochromator: graphite	*R*_int_ = 0.025
*T* = 273(2) K	θ_max_ = 25.5º
φ and ω scans	θ_min_ = 1.9º
Absorption correction: multi-scan(SADABS; Sheldrick, 1996)	*h* = −8→8
*T*_min_ = 0.035, *T*_max_ = 0.134	*k* = −10→10
6783 measured reflections	*l* = −13→13

### Refinement

**Table:**

Refinement on *F*^2^	Secondary atom site location: difference Fourier map
Least-squares matrix: full	Hydrogen site location: inferred from neighbouring sites
*R*[*F*^2^ > 2σ(*F*^2^)] = 0.018	H-atom parameters constrained
*wR*(*F*^2^) = 0.043	*w* = 1/[σ^2^(*F*_o_^2^) + (0.0209*P*)^2^ + 0.4377*P*] where *P* = (*F*_o_^2^ + 2*F*_c_^2^)/3
*S* = 1.05	(Δ/σ)_max_ = 0.002
2544 reflections	Δρ_max_ = 0.89 e Å^−^^3^
173 parameters	Δρ_min_ = −0.89 e Å^−^^3^
Primary atom site location: structure-invariant direct methods	Extinction correction: none

### Special details

**Table:**

Geometry. All e.s.d.'s (except the e.s.d. in the dihedral angle between two l.s. planes) are estimated using the full covariance matrix. The cell e.s.d.'s are taken into account individually in the estimation of e.s.d.'s in distances, angles and torsion angles; correlations between e.s.d.'s in cell parameters are only used when they are defined by crystal symmetry. An approximate (isotropic) treatment of cell e.s.d.'s is used for estimating e.s.d.'s involving l.s. planes.
Refinement. Refinement of *F*^2^ against ALL reflections. The weighted *R*-factor *wR* and goodness of fit *S* are based on *F*^2^, conventional *R*-factors *R* are based on *F*, with *F* set to zero for negative *F*^2^. The threshold expression of *F*^2^ > σ(*F*^2^) is used only for calculating *R*-factors(gt) *etc*. and is not relevant to the choice of reflections for refinement. *R*-factors based on *F*^2^ are statistically about twice as large as those based on *F*, and *R*- factors based on ALL data will be even larger.

### Fractional atomic coordinates and isotropic or equivalent isotropic displacement parameters (Å^2^)

**Table:**

	*x*	*y*	*z*	*U*_iso_*/*U*_eq_	
C8	0.3562 (5)	0.2238 (4)	−0.1887 (4)	0.0399 (8)	
H8	0.3666	0.1521	−0.1411	0.048*	
C9	0.3852 (5)	0.1810 (4)	−0.3149 (4)	0.0432 (9)	
H9	0.4144	0.0802	−0.3542	0.052*	
Pt1	0.216802 (16)	0.685427 (14)	−0.091569 (11)	0.03068 (6)	
Cl2	0.40740 (17)	0.23872 (14)	−0.54110 (10)	0.0599 (3)	
Cl1	0.19823 (17)	0.81788 (12)	−0.24323 (10)	0.0529 (3)	
N1	0.2327 (4)	0.5583 (4)	0.0285 (3)	0.0311 (6)	
N2	0.2763 (4)	0.4162 (3)	−0.0062 (3)	0.0356 (6)	
C1	0.1910 (4)	0.6209 (4)	0.1559 (3)	0.0322 (7)	
C7	0.3111 (4)	0.3755 (4)	−0.1328 (3)	0.0329 (7)	
C10	0.3698 (5)	0.2924 (4)	−0.3816 (3)	0.0390 (8)	
C2	0.1378 (5)	0.7727 (4)	0.1893 (3)	0.0347 (7)	
C11	0.3267 (5)	0.4437 (4)	−0.3279 (3)	0.0370 (8)	
H11	0.3186	0.5149	−0.3763	0.044*	
C5	0.1648 (6)	0.6038 (5)	0.3660 (4)	0.0480 (10)	
H5	0.1715	0.5467	0.4254	0.058*	
S1	0.10822 (13)	0.87598 (11)	0.07262 (8)	0.0362 (2)	
C12	0.2953 (5)	0.4887 (4)	−0.2005 (3)	0.0326 (7)	
C4	0.1152 (6)	0.7564 (6)	0.4010 (4)	0.0515 (10)	
H4	0.0924	0.8021	0.4841	0.062*	
C6	0.2041 (5)	0.5359 (5)	0.2441 (4)	0.0412 (8)	
H6	0.2389	0.4342	0.2213	0.049*	
C3	0.0998 (5)	0.8407 (5)	0.3127 (4)	0.0454 (9)	
H3	0.0643	0.9421	0.3358	0.054*	
C13	0.2718 (6)	1.0531 (5)	0.1290 (4)	0.0519 (10)	
H13A	0.2399	1.1140	0.2093	0.078*	
H13B	0.2714	1.1164	0.0704	0.078*	
H13C	0.3908	1.0227	0.1371	0.078*	

### Atomic displacement parameters (Å^2^)

**Table:**

	*U*^11^	*U*^22^	*U*^33^	*U*^12^	*U*^13^	*U*^23^
C8	0.041 (2)	0.0332 (19)	0.048 (2)	0.0096 (15)	0.0056 (17)	0.0131 (17)
C9	0.043 (2)	0.0313 (19)	0.052 (2)	0.0099 (15)	0.0065 (18)	0.0042 (17)
Pt1	0.03822 (9)	0.02666 (9)	0.02684 (9)	0.00541 (6)	0.00145 (6)	0.00626 (6)
Cl2	0.0827 (8)	0.0549 (6)	0.0349 (5)	0.0134 (6)	0.0109 (5)	−0.0030 (5)
Cl1	0.0879 (8)	0.0395 (5)	0.0383 (5)	0.0191 (5)	0.0114 (5)	0.0174 (4)
N1	0.0315 (15)	0.0336 (16)	0.0279 (15)	0.0057 (12)	0.0014 (12)	0.0074 (12)
N2	0.0366 (15)	0.0343 (16)	0.0374 (17)	0.0074 (12)	0.0041 (13)	0.0112 (13)
C1	0.0314 (17)	0.0361 (18)	0.0274 (17)	0.0024 (14)	−0.0006 (13)	0.0069 (14)
C7	0.0318 (17)	0.0315 (18)	0.0350 (19)	0.0042 (14)	0.0018 (14)	0.0082 (15)
C10	0.0388 (19)	0.040 (2)	0.0324 (19)	0.0039 (15)	0.0053 (15)	0.0006 (16)
C2	0.0356 (18)	0.0370 (19)	0.0294 (18)	0.0031 (14)	0.0004 (14)	0.0065 (15)
C11	0.044 (2)	0.0319 (18)	0.0340 (19)	0.0031 (15)	0.0027 (16)	0.0081 (15)
C5	0.052 (2)	0.063 (3)	0.033 (2)	0.006 (2)	−0.0015 (17)	0.0205 (19)
S1	0.0434 (5)	0.0343 (5)	0.0302 (5)	0.0113 (4)	0.0005 (4)	0.0054 (4)
C12	0.0334 (17)	0.0279 (17)	0.0330 (19)	0.0020 (13)	−0.0012 (14)	0.0033 (14)
C4	0.049 (2)	0.073 (3)	0.031 (2)	0.008 (2)	0.0055 (17)	0.011 (2)
C6	0.043 (2)	0.046 (2)	0.037 (2)	0.0049 (16)	−0.0015 (16)	0.0164 (17)
C3	0.048 (2)	0.053 (2)	0.034 (2)	0.0100 (18)	0.0056 (17)	0.0071 (18)
C13	0.070 (3)	0.034 (2)	0.047 (2)	0.0003 (19)	−0.005 (2)	0.0070 (18)

### Geometric parameters (Å, °)

**Table:**

C8—C9	1.378 (6)	C10—C11	1.382 (5)
C8—C7	1.394 (5)	C2—C3	1.390 (5)
C8—H8	0.9300	C2—S1	1.785 (4)
C9—C10	1.385 (6)	C11—C12	1.395 (5)
C9—H9	0.9300	C11—H11	0.9300
Pt1—N1	1.959 (3)	C5—C6	1.378 (6)
Pt1—C12	1.986 (3)	C5—C4	1.388 (6)
Pt1—Cl1	2.2911 (11)	C5—H5	0.9300
Pt1—S1	2.3529 (10)	S1—C13	1.811 (4)
Cl2—C10	1.742 (4)	C4—C3	1.383 (6)
N1—N2	1.286 (4)	C4—H4	0.9300
N1—C1	1.429 (4)	C6—H6	0.9300
N2—C7	1.391 (5)	C3—H3	0.9300
C1—C6	1.385 (5)	C13—H13A	0.9600
C1—C2	1.391 (5)	C13—H13B	0.9600
C7—C12	1.407 (5)	C13—H13C	0.9600
			
C9—C8—C7	119.5 (4)	C10—C11—C12	119.4 (3)
C9—C8—H8	120.3	C10—C11—H11	120.3
C7—C8—H8	120.3	C12—C11—H11	120.3
C8—C9—C10	118.2 (3)	C6—C5—C4	120.7 (4)
C8—C9—H9	120.9	C6—C5—H5	119.7
C10—C9—H9	120.9	C4—C5—H5	119.7
N1—Pt1—C12	79.26 (14)	C2—S1—C13	102.24 (19)
N1—Pt1—Cl1	175.94 (9)	C2—S1—Pt1	95.61 (12)
C12—Pt1—Cl1	96.84 (11)	C13—S1—Pt1	111.80 (16)
N1—Pt1—S1	86.25 (9)	C11—C12—C7	117.3 (3)
C12—Pt1—S1	165.07 (11)	C11—C12—Pt1	131.9 (3)
Cl1—Pt1—S1	97.57 (4)	C7—C12—Pt1	110.7 (3)
N2—N1—C1	118.9 (3)	C3—C4—C5	120.2 (4)
N2—N1—Pt1	120.9 (2)	C3—C4—H4	119.9
C1—N1—Pt1	120.2 (2)	C5—C4—H4	119.9
N1—N2—C7	110.8 (3)	C5—C6—C1	119.2 (4)
C6—C1—C2	120.7 (3)	C5—C6—H6	120.4
C6—C1—N1	121.9 (3)	C1—C6—H6	120.4
C2—C1—N1	117.4 (3)	C4—C3—C2	119.5 (4)
N2—C7—C8	119.2 (3)	C4—C3—H3	120.2
N2—C7—C12	118.3 (3)	C2—C3—H3	120.2
C8—C7—C12	122.4 (3)	S1—C13—H13A	109.5
C11—C10—C9	123.3 (3)	S1—C13—H13B	109.5
C11—C10—Cl2	118.5 (3)	H13A—C13—H13B	109.5
C9—C10—Cl2	118.2 (3)	S1—C13—H13C	109.5
C3—C2—C1	119.7 (3)	H13A—C13—H13C	109.5
C3—C2—S1	120.1 (3)	H13B—C13—H13C	109.5
C1—C2—S1	120.1 (3)		
			
C7—C8—C9—C10	0.6 (6)	C1—C2—S1—Pt1	−6.0 (3)
C12—Pt1—N1—N2	−1.8 (3)	N1—Pt1—S1—C2	4.51 (14)
S1—Pt1—N1—N2	174.6 (3)	C12—Pt1—S1—C2	18.4 (4)
C12—Pt1—N1—C1	179.9 (3)	Cl1—Pt1—S1—C2	−176.89 (12)
S1—Pt1—N1—C1	−3.7 (2)	N1—Pt1—S1—C13	110.10 (18)
C1—N1—N2—C7	179.6 (3)	C12—Pt1—S1—C13	124.0 (4)
Pt1—N1—N2—C7	1.4 (4)	Cl1—Pt1—S1—C13	−71.30 (16)
N2—N1—C1—C6	3.0 (5)	C10—C11—C12—C7	0.5 (5)
Pt1—N1—C1—C6	−178.7 (3)	C10—C11—C12—Pt1	−175.7 (3)
N2—N1—C1—C2	−177.6 (3)	N2—C7—C12—C11	−178.5 (3)
Pt1—N1—C1—C2	0.6 (4)	C8—C7—C12—C11	0.1 (5)
N1—N2—C7—C8	−178.4 (3)	N2—C7—C12—Pt1	−1.6 (4)
N1—N2—C7—C12	0.2 (4)	C8—C7—C12—Pt1	177.0 (3)
C9—C8—C7—N2	178.0 (3)	N1—Pt1—C12—C11	178.0 (4)
C9—C8—C7—C12	−0.6 (5)	Cl1—Pt1—C12—C11	−0.8 (3)
C8—C9—C10—C11	0.0 (6)	S1—Pt1—C12—C11	163.9 (2)
C8—C9—C10—Cl2	179.7 (3)	N1—Pt1—C12—C7	1.7 (2)
C6—C1—C2—C3	0.9 (5)	Cl1—Pt1—C12—C7	−177.2 (2)
N1—C1—C2—C3	−178.5 (3)	S1—Pt1—C12—C7	−12.4 (6)
C6—C1—C2—S1	−176.2 (3)	C6—C5—C4—C3	1.8 (6)
N1—C1—C2—S1	4.4 (4)	C4—C5—C6—C1	−1.0 (6)
C9—C10—C11—C12	−0.5 (6)	C2—C1—C6—C5	−0.4 (5)
Cl2—C10—C11—C12	179.7 (3)	N1—C1—C6—C5	179.0 (3)
C3—C2—S1—C13	63.1 (4)	C5—C4—C3—C2	−1.3 (6)
C1—C2—S1—C13	−119.8 (3)	C1—C2—C3—C4	−0.1 (6)
C3—C2—S1—Pt1	176.9 (3)	S1—C2—C3—C4	177.0 (3)
